# Retarding breast tumor growth with nanoparticle-facilitated intravenous delivery of BRCA1 and BRCA2 tumor suppressor genes

**DOI:** 10.1038/s41598-022-25511-9

**Published:** 2023-01-11

**Authors:** Nabilah Ibnat, Ezharul Hoque Chowdhury

**Affiliations:** 1 Jeffrey Cheah School of Medicine and Health Sciences, Monash University Malaysia, Selangor, Malaysia; 2 Department of Bioengineering, George Mason University, Fairfax, VA 20110 USA

**Keywords:** Breast cancer, Drug delivery

## Abstract

Gene augmentation therapy entails replacement of the abnormal tumor suppressor genes in cancer cells. In this study, we performed gene augmentation for BRCA1/2 tumor suppressors in order to retard tumor development in breast cancer mouse model. We formulated inorganic carbonate apatite (CA) nanoparticles (NPs) to carry and deliver the purified BRCA1/2 gene- bearing plasmid DNA both in vitro and in vivo. The outcome of BRCA1/2 plasmid-loaded NPs delivery on cellular viability of three breast cancer cell lines such as MCF-7, MDA-MB-231 and 4T1 were evaluated by MTT assay. The result in MCF-7 cell line exhibited that transfection of BRCA 1/2 plasmids with CA NPs significantly reduced cancer cell growth in comparison to control group. Moreover, we noticed a likely pattern of cellular cytotoxicity in 4T1 murine cancer cell line. Following transfection with BRCA1 plasmid-loaded NPs, and Western blot analysis, a notable reduction in the phospho-MAPK protein of MAPK signaling pathway was detected, revealing reduced growth signal. Furthermore, in vivo study in 4T1 induced breast cancer mouse model showed that the tumor growth rate and final volume were decreased significantly in the mouse group treated intravenously with BRCA1 + NPs and BRCA2 + NPs formulations. Our results established that BRCA1/2 plasmids incorporated into CA NPs mitigated breast tumor growth, signifying their application in the therapy for breast cancer.

## Introduction

As reported by ‘Global Cancer Statistics 2020’, breast cancer has exceeded lung cancer being the most frequently diagnosed cancer among women, with a projected 2.3 million new cases (11.7%)^1^. The incidence of cases varied worldwide, with rates extending from 28 per 100,000 in South East Asia to 127 in the United States of America^2^. Yet, more female breast cancer patients were losing their lives in developing versus developed countries (15.0 vs 12.8 every 100,000)^1^.

Currently, a number of treatment options are available against breast cancer such as surgery, radiotherapy, chemotherapy, endocrine therapy, immunotherapy and targeted therapy depending on location of metastases and morphological features. Although technical and scientific research paves the way for upgraded therapies, the first-line treatments effectiveness can’t be certain, and might also cause long-term side effects, as well as nausea, weight loss, hair loss etc.^3,4^. Based on gene expression characteristics, different molecular subtypes of breast cancer require different types of systematic treatments^5,6^. In the MINDACT trial, Cardoso et. al showed that tumors with “low risk” genomic features do not need chemotherapy^7^. However, the TAILORx study revealed that breast cancer detected at early stage, with node-negative feature did not respond well from adjuvant chemotherapy, however treatment with endocrine therapy revealed promising outcome^8^. Intrinsically, breast cancer is categorized by multiple molecular genetic mutations and hence clinically explored for gene therapy interventions^9^. Current knowledge of tumor suppresser genes in the genesis of breast malignancy has moved the development of gene therapy strategies directed at restoring such genes. Several tumor suppressors such as p53, PTEN, BRCA1, and BRCA2 have been reported to be mutated in breast cancer, whose function can be restored by gene augmentation therapy. In gene augmentation therapy, therapeutic DNA containing a functional copy of the selected gene (i.e. plasmid) is introduced into the cell to restore the role of the lost genes^10^.

*BRCA1* and *BRCA2* (breast cancer type 1 and 2) *are* tumor suppressor proteins, typically expressed in the breast and other tissues of female organ, where they assist repairing injured DNA, or abolish cells if DNA can’t be repaired^11^. Mutations in BRCA1 and BRCA2 possess a higher risk of developing breast cancer in women. Females carrying mutations in BRCA1, have about 57–65% of possibility of developing breast cancer during their lifetime^12^. Germ-line mutations in BRCA1 gene are related to about 25% of familial breast cancer, while somatic inactivation of BRCA1 is detected in up to 5% of sporadic breast cancers^13^. Basically, a highly aggressive, metastatic subtype is the Triple Negative Breast Cancer (TNBC) which lacks expression of estrogen alpha receptor (ERα), progesterone, and ERBB2 receptors and comprises nearly 15% of breast cancer cases. Among the TNBC patients, nearly 70% of tumors contain germline mutations in BRCA1 gene^14,15^. In addition, BRCA2 germ-line mutations occur in about 35% of women with early-onset breast cancer^16^. Breast tumors with BRCA-mutations showed higher clinical grade and stage of the disease with enhanced metastatic capacity^17^.

The main function of BRCA1 is to repair DNA damage, largely of double-stranded DNA breaks (DSBs). Upon a DNA damage response, BRCA1 is employed at DNA damage sites, and assists as a binding framework for other DNA repair proteins^13^, eventually allowing the assembling of RAD51 recombinase and supporting the homologous recombination-mediated (HR) DNA repair^18^. However, BRCA1 and BRCA2 play different roles in DSB repair by HR. Existing result suggested a direct involvement for BRCA2 in this mechanism. Research by Davis et al. showed that intracellular transport and activity of RAD51 was regulated by BRCA2^19^. Furthermore, BRCA1 and BRCA2 in the cytosol take part in regulating numerous molecular mechanisms during mitosis of cell division. Mutations in BRCA1/2 definitely impact their significant functions and can harm the stability of the highly regulated cellular processes, leading to the growth of cancer^14^.

As a consequence, considering the important roles of BRCA1 and BRCA2 mutations in development of breast cancer, strategies in supplying the wild type BRCA1 and BRCA2 gene could be promising as a therapeutic option. However, the bottleneck in producing gene therapy-based medicines relies on the delivery of the genetic material. Since DNA is -susceptible cleavage by circulating nucleases; therefore, various strategies have been deployed to protect it and assist with its transportation into cell^9^. In this study, we focused on BRCA1 and BRCA2 gene delivery with a view to reduce breast cancer cell proliferation in different breast cancer cells. We designed our study to deliver BRCA1 and BRCA2 tumor suppressors in the form of plasmid by means of inorganic Carbonate Apatite (CA) nanoparticles (NPs) carrier and evaluated breast cancer cell growth both in vitro and in vivo.

Chowdhury et al. reported biodegradable smart pH-sensitive CA NPs which deliver the nanoparticle bound plasmid DNA in the cell and eventually release the DNA carrying specific gene from endosomes to cytosol, facilitating expression of the gene of interest inserted onto the plasmid^20^. Since CA NP is pH responsive, studies showed the effect of pH in regulation of particle synthesis, endosomal escape and transfection efficiency^21,22^. At pH 7.4, addition of Ca^2+^ salt and plasmid DNA to bicarbonate-buffered medium forms nano sized CA-DNA complexes, when incubated at 37 °C for 30 min. Subsequently, addition of FBS stops formation of further nanoparticle complex in the media^22^. CA NPs with a molecular formula of Ca_10_ (PO_4_)_6−x_ (CO_3_)_x_ (OH)_2,_ show the average diameter of 50–300 nm with strong binding affinity to the plasmid DNA^21,23^. The positively charged Ca^2+^ in the CA NPs can electrostatically associate with the negatively charged plasmid DNA molecules and the resulting complex crosses the cell membrane via endocytosis^22^. At endosomal acidic pH, particles are rapidly degraded into the constituent ions such as Ca^2+^, PO_4_^3−^ and HCO_3_^−^, resulting in nano particle dissolution, rupture of the endosomes and finally release of the DNA in cytosol^22,23^.

Next, released plasmids in the cytosol are subject to immediate interaction with several microtubule proteins, for example, kinesin and dynein. Binding of the plasmid DNA with these proteins mediates its translocation to the nucleus via the nuclear pore or during cell division, encouraging transgene expression in the cell^24^. These NPs exhibit low immunogenicity and toxicity profile in animal model and ensure extended plasma half-life in circulation and greater accumulation of the nucleic acids in the tumor site, enabling their effective cellular uptake through endocytosis and fast intracellular release into cytoplasm^22^. Our study here demonstrated the primary use of biodegradable CA NPs to deliver BRCA1 and BRCA2 plasmids into breast cancer cells and explored the outcome of gene augmentation on tumor regression.

## Results

### Size and zeta potential of BRCA1 and BRCA2 plasmids-loaded NPs

**Figure 1:**
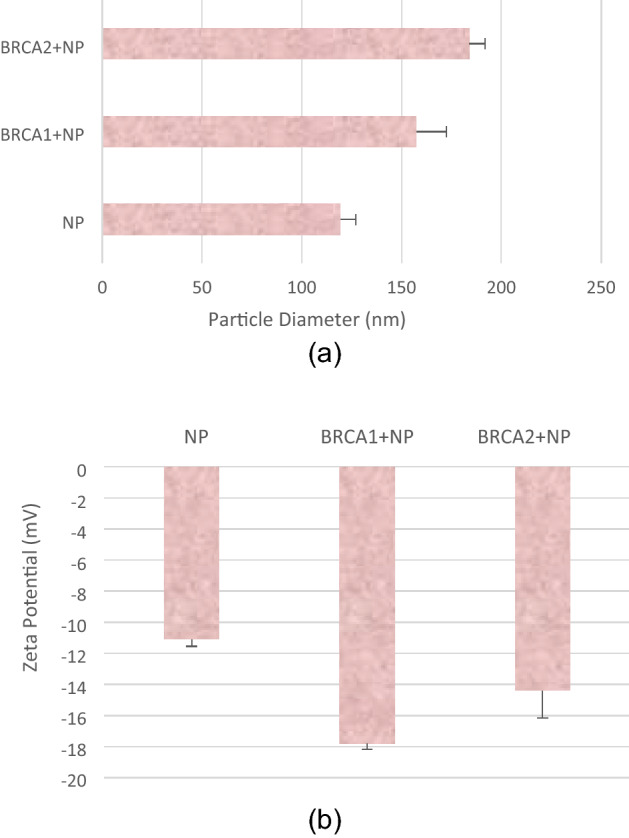
**(a)** Size and **(b)** zeta potential measurement. NPs, BRCA1 + NPs, and BRCA2 + NPs formed with addition of 4 mM of exogeneous Ca^2+^ in 1 mL DMEM medium with 1 µg of BRCA1 or BRCA2 plasmid DNA, followed by incubation for 30 min at 37 °C. Each of the measurements was performed three times and mean and standard deviation were calculated.

The size and zeta potential of BRCA1 and BRCA2 plasmids (1 µg of each plasmid DNA) in association with CA NP formulations prepared with 4 mM CaCl_2_, were presented in Fig. 1 (a, b). From Fig. 1 (a), it was observed that the diameter of the CA NP only was ~ 119 nm, while the particle diameter following addition of BRCA1 and BRCA2 plasmids increased to ~ 157 nm and ~ 184 nm, respectively. As represented in Fig. 1 (b), the zeta potential of NP control (formulated with 4 mM CaCl_2_) was detected to be approximately -11 mV. The surface charge of the plasmid + NP complexes was more electronegative due to incorporation of negatively charged plasmid DNA. The zeta potentials for BRCA1 + NP and BRCA2 + NP complexes were recorded as approximately − 18 mV and − 14 mV, respectively. This is beneficial since the tendency to cluster with the anionic serum proteins in the cytoplasm would be prohibited due to the negative zeta potential of the plasmid-loaded NPs.

### Assessment of cell viability of breast cancer cells treated with NPs carrying BRCA1 and BRCA2 tumor suppressor plasmid genes

Transgene expression of BRCA1 and BRCA2 in breast cancer cell lines using the novel vector of CA NPs potentially provided new insight for breast cancer gene therapy. The cell proliferation rate measured by the MTT assay was used to investigate the cell viability of the plasmid treatment in three types of breast cancer cell lines.

**Figure 2:**
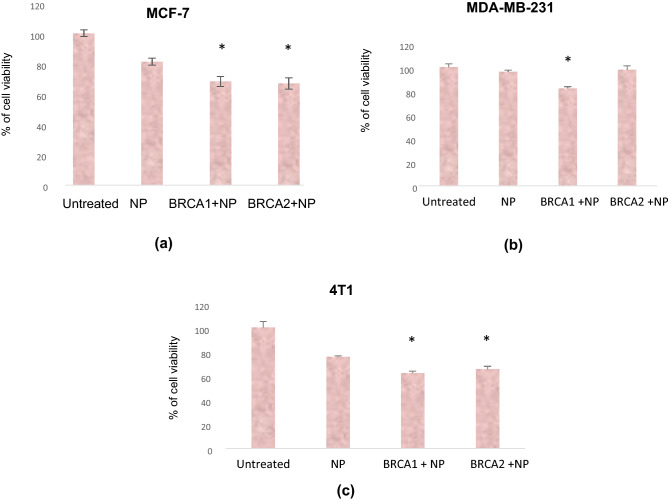
Cell viability assessment of BRCA1 and BRCA2 plasmid delivery in **(a)** MCF-7 **(b)** MDA-MB-231 and **(c)** 4T1 cells treated with BRCA1 + NP, and BRCA2 + NP formulations for 48 h. The experiments were performed three times in each of the cell lines and values were presented as mean ± SD of triplicates in MTT assay. * indicated *p* < 0.05 compared to NPs control.

We selected MCF-7, the estrogen receptor positive cell line and treated with NPs, 1 µg BRCA1 + NPs and 1 µg BRCA2 + NP complexes for 48 h. As shown in Fig. 2 (a), NP treatment exhibited ~ 81% of cell viability in comparison to the untreated cells indicating ~ 19% cytotoxicity effect due to CA NPs. Treatment with BRCA1 + NP and BRCA2 + NP complexes of 1 µg plasmids reduced cell viability to ~ 68% and ~ 67%, respectively. Thus, compared to NP-treated cells, actual cytotoxicity effect of BRCA1 + NP and BRCA2 + NP formulations were calculated to be 13% and 14%, respectively.

The second type of cell line was MDA-MB-231, the triple-negative breast cancer type, one of the most aggressive form with very few treatment opportunities available. For BRCA1/BRCA2 transgene expression we prepared the NP-plasmid complexes and treated the cells with NP, BRCA1 + NP and BRCA2 + NP formulations for 48 h. In Fig. 2 (b), NP assisted delivery of BRCA1, declined the cell viability to ~ 82% (i.e. ~ 14% actual cytotoxicity) while, BRCA2 + NP formulation apparently didn’t exert any cytotoxicity.

In gene therapy, the plasmid DNA size is a key factor for its successful translocation into the cell nucleus. In our experiment, the size of BRCA1 plasmid was 5600 bp; thus, it might enter the nucleus during the cell division process. However, the large size (10 Kbp) of BRCA2 plasmid might entail some barriers when incorporated with CA NPs and couldn’t efficiently be transported to the nucleus across the nuclear pore in this particular cell line.

The 4T1 is a murine mammary gland carcinoma cell line which is transplantable, highly tumorigenic and can spontaneously metastasize. From Fig. 2 (c), in 4T1 cells, the BRCA1 + NP formulation exhibited ~ 13% cytotoxicity and BRCA2 + NP treatment showed ~ 10% individual cytotoxicity, suggesting both BRCA1 and BRCA2 genes expression in the cell line which participated in enhancing the cytotoxicity effect of the treatments. The cytotoxicity data in all three cell lines for BRCA1 + NP and BRCA2 + NP treatment is summarized as tabulated form (Table 1).

**Table 1:** Cytotoxicity imposed by BRCA1 + NP and BRCA2 + NP formulations in three breast cancer cell lines.

Cell line	Treatment	Actual Cytotoxicity
MCF-7	BRCA1 + NP	~ 13%
	BRCA2 + NP	~ 14%
MDA-MB-231	BRCA1 + NP	~ 14%
	BRCA2 + NP	~ − 2%
4T1	BRCA1 + NP	~ 13%
	BRCA2 + NP	~ 10%

### Observation of treated cells under the light microscope

**Figure 3:**
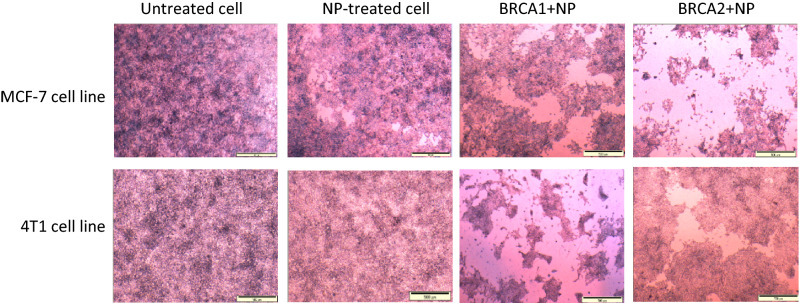
Light microscopic images of two different cell lines following treatment with NP, BRCA1 + NP and BRCA2 + NP. Cells treated with BRCA1 + NP and BRCA2 + NP, revealed decrease in cellular density in MCF-7 (upper panel) and 4T1 (lower panel) cell line, compared to the untreated control cells (concentrated).

Treatment of MCF-7 and 4T1 cells with 4 mM NP, BRCA1 + NP and BRCA2 + NP for 48 h induced morphological changes in cell size and shape and was observed under light microscope. The images in Fig. 3 demonstrated that untreated control cells are packed within 48 h of incubation, while in presence of 4 mM Ca^2+^, the cells appear to be surrounded by the CA NPs, indicating that particles formulation could lead to minor cytotoxicity. In addition, microscopic observation of the cells treated with BRCA1 + NP and BRCA2 + NP, revealed decrease in cellular density in MCF-7 (upper panel) and 4T1 (lower panel) cell line, compared to the untreated control cells (concentrated). This simple visualization of treated cells with less cell number indicated cell shrinkage and reduced cellular proliferation which was also reflected in the cytotoxicity results performed earlier.

### Modulation of phospho-MAPK protein expression in the MAPK signaling pathway following treatment with BRCA1 + NP and BRCA2 + NP complexes

We explored the effects of BRCA1 + NP and BRCA2 + NP on decreasing cell viability in MCF-7 cell line, by analyzing the expression level of activated phospho-MAPK (responsible for proliferative signal) and total MAPK using Western blot and densitometry.

**Figure 4:**
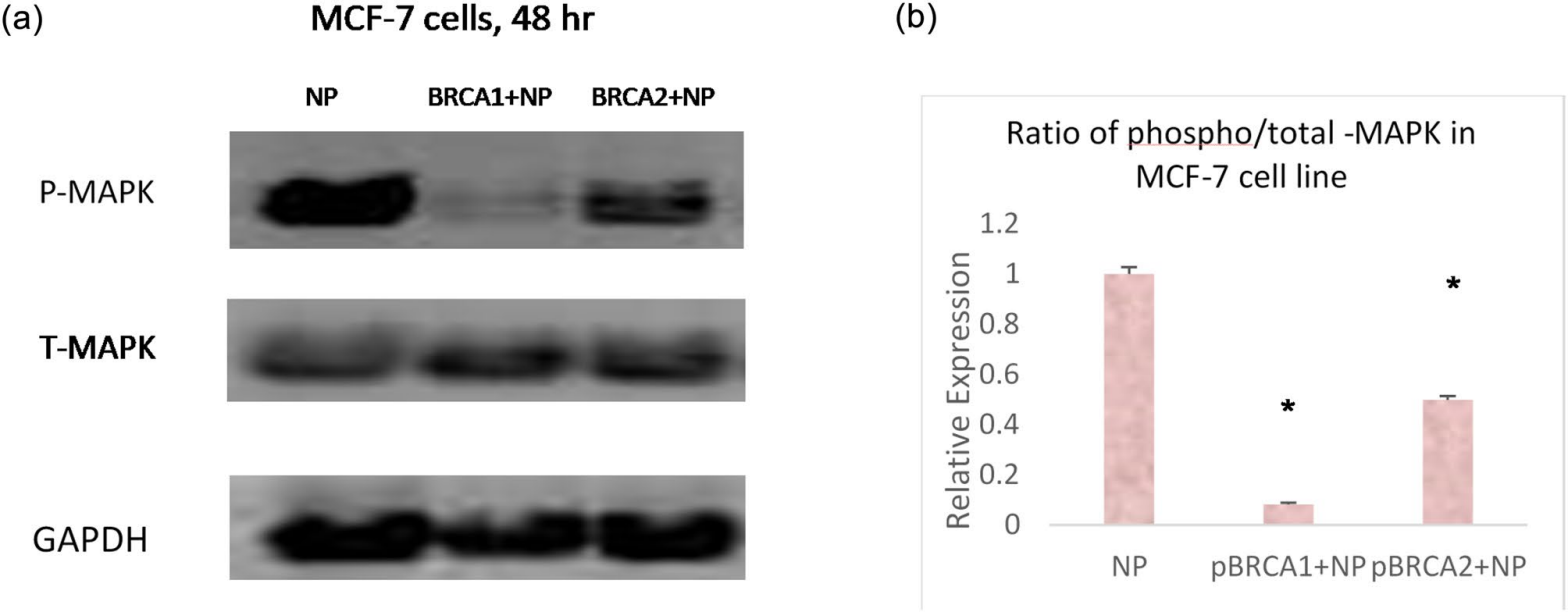
**(a)** Western Blot image following intracellular delivery of BRCA1 + NP and BRCA2 + NP in MCF-7 cell line. Cells were incubated with NPs with loaded BRCA1 and BRCA2 plasmids for 48 h prior to cell lysis for protein extraction and Western blotting analysis. Detection of P-MAPK, T-MAPK and GAPDH (housekeeping protein) was performed using respective primary antibodies. **(b)** Densitometry analysis showing ratio of P-MAPK/total MAPK expression. * indicates *p* < 0.05, with significant change in protein expression profile.

Treatment of MCF-7 breast cancer cells with BRCA1 + NP and BRCA2 + NP was linked with de-phosphorylation (deactivated form) of MAPK without an alteration in the total MAPK level (Fig. 4a). As shown in Fig. 4 (b), the ratio calculated for phospho-MAPK to total MAPK protein expression is very significant for BRCA1 + NP treatment, and for BRCA2 + NP formulation. Less expression of phospho-MAPK protein referred to diminished signal for cellular proliferation, which is also correlated with the in vitro cytotoxicity results.

### Effect of NP-facilitated BRCA1 and BRCA2 gene delivery on tumor regression profile

**Figure 5:**
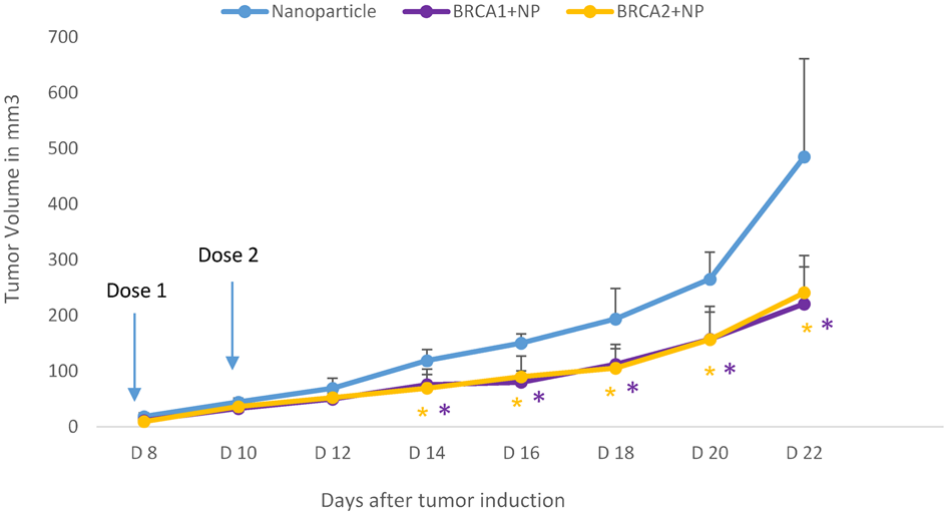
Tumor regression study following intravenous delivery of NP, BRCA1 + NP, and BRCA2 + NP in 4T1 cells-induced tumor mouse model. 4T1 cells were inoculated subcutaneously on the mammary pad of mice. On day 8, as the tumor reached an approximate volume of ~ 25 mm^3^, mice were treated intravenously through tail-vein injection with 100 μL of NP (8 M Ca^2+^), BRCA1 + NP (40 µg of plasmid) and BRCA2 + NP (40 µg of plasmid); followed by the 2nd dose at day 10. The tumor outgrowth was monitored until day 22. Five mice were used per group and data were represented as mean ± SD. Compared to NP control group, statistical analysis was significant, * when *p* < 0.05. (* *p* < 0.05 (NP control and BRCA1 + NP), * *p* < 0.05 (NP control and BRCA2 + NP).

We induced breast tumors in Balb/c mice and tested the formulations (NP, BRCA1 + NP and BRCA2 + NP) in vivo. As shown in Fig. 5, following intravenous delivery, compared to NP only control group, mice treated with BRCA1 plasmid + NP exhibited reduced trend for development of tumor and the difference was significant at day 14, day 16, day 18, day 20 and day 22; where *p < 0.05.

This result suggested effective delivery of BRCA1 gene in mice breast tumors with the help of CA NP, and displayed significant tumor regression from day 14 onwards. Also, in comparison to NP control group, reduced tumor development was observed in mice treated with BRCA2 + NP formulation where the *p*-value was significant at day 14, day 16, day 18, day 20 and day 22. Such tumor regression profile also indicated BRCA2 delivery and expression in the tumor tissue which might suppress the growth of 4T1 cancer cells in mice.

Also, none of the mice from any treatment groups presented any change in behavior after the intravenous injections and their body weight persisted almost the same for each group of mice tested (data not provided).

### Quantitation of tumor volume and weight upon treatment

**Figure 6:**
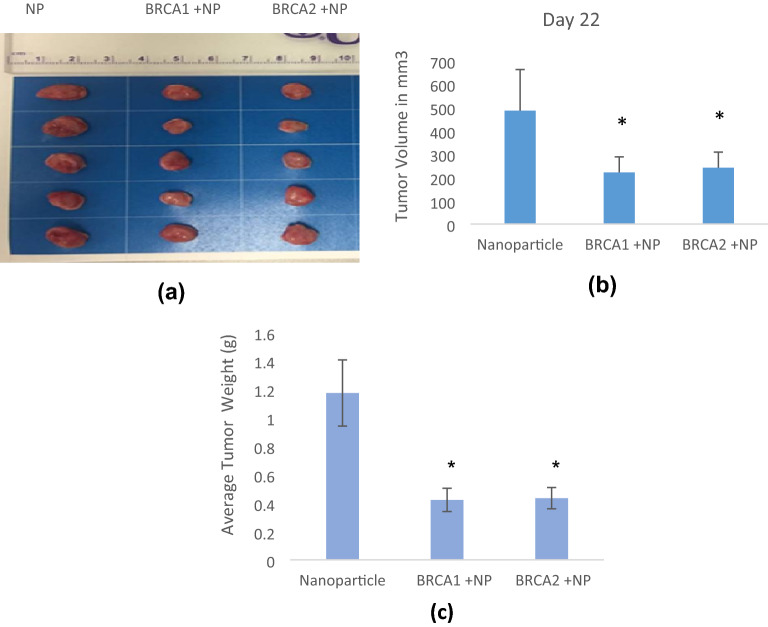
Transgene delivery of BRCA1 + NP and BRCA2 + NP in 4T1-induced tumor mouse model inhibited tumor growth. (**a**) Tumor images captured following sacrifice at day 22. (**b**) Quantitation of tumor volumes in NP control, BRCA1 plasmid-treated mice and BRCA2 plasmid-treated mice. (**c**) Quantitation of tumor weights. Statistical analysis was found significant compared to NP control group, * when *p* < 0.05.

As presented in Fig. 6 (a,b), upon sacrifice of mice at day 22, BRCA1 + NP treated mice displayed significant reduction in tumor volume in comparison to the NP control group (~ 221 mm^3^ vs. 485 mm^3^, p < 0.05). Additionally, BRCA2 + NP treated mice showed lesser tumor volume than the control (~ 241 mm^3^ vs. 485 mm^3^, *p* < 0.05). Hence, both treatment groups exhibited reduced tumor volumes, which implied importance of BRCA1 and BRCA2 proteins as tumor suppressors p play role on tumor volume reduction. As shown in Fig. 6 (c), the tumor weight in BRCA1 + NP treated mice group was lower compared to the NP control group (0.4208 g vs. 1.172 g, *p* < 0.05). In addition, we demonstrated that the tumor weight in BRCA2 + NP treated mice was also reduced (0.4332 g vs. 1.172 g, *p* < 0.05).

## Discussion

Screening for mutations in BRCA 1/2 genes for predictive genetic testing has become widespread, and women having such mutations are subsequently found to undergo risk reducing mastectomy or oophorectomy^25^, that can significantly decrease morbidity and mortality. However, in recent years molecularly targeted therapeutics received FDA approval for the treatment of HER2-negative breast cancer bearing inherited BRCA1/2 mutation^26^.

Germline mutations in the BRCA tumor suppressor genes possess approximately 45–65% risk of developing breast cancer and a 17–39% risk of ovarian cancer over the lifetime^14,27^. In such patients, in order to repair DNA damage, breast cells carrying BRCA1/2 mutation depend on an alternative pathway called base excision repair pathway in presence of poly ADP-ribose polymerase (PARP) proteins. PARP plays a vital role in the repair of single-strand DNA breaks. However, PARP inhibitors function to persuade piling up of single-strand DNA damage and can produce double-strand breaks (DSB). DSB normally get repaired via homologous recombination repair (HRR), a complex process requiring many proteins, especially BRCA1 and BRCA2. In tumors with BRCA1/2 mutation, lacking HRR, an alternative DNA repair by nonhomologous end joining (a low fidelity repair mechanism), results in cell death^28^. In this regard, using PARP inhibitors prevent cancer cells from repairing, allowing them to die. The first randomized phase III OlympiAD clinical trial tested a PARP inhibitor, olaparib for breast cancer patients harboring an inherited BRCA1/2 mutation^29^. In comparison to standard therapy, olaparib monotherapy showed a significant benefit among patients with a germline BRCA mutation having HER2-negative metastatic breast cancer. Also reported that the median progression-free survival was 2.8 months longer and the death risk was 42% reduced with olaparib monotherapy^30^.

In addition, other treatments target the BRCA1 gene based on the inverse correlation between expression of BRCA1 and the occurrence of breast cancer^31,32^. Several studies observed that most sporadic breast and ovarian cancers showed low levels of *BRCA1* messenger RNA (mRNA) and protein expression^33,34^. Loss of heterozygosity and promoter methylation of the remaining *BRCA1* allele might cause the lower *BRCA1* mRNA^33,35,36^ indicating that restoration of ‘wild-type’ *BRCA1 gene* expression may impede tumors by a ‘genetic correction’ approach, in many sporadic cancers. In this context, quite a few group of researchers proved that expression of the BRCA1 gene leads to decreased growth and/or cell death, signifying that BRCA1 can take part as a tumor suppressor^33,37,38^. Experiments in nude mice xenografts demonstrated that intraperitoneal injection of retroviral vectors expressing BRCA1sv (splice variant of BRCA1) could prevent the proliferation of intraperitoneal tumors, compared to control retroviral vectors^35^. Also, Holt et al. showed that retroviral transfer of the wild-type *BRCA1* gene hindered growth of all breast and ovarian cancer cell lines tested, in vitro^37^. Moreover, there are evidences that apoptosis or cell cycle arrest at the G2/M stage in various cell types could be induced by overexpression of BRCA1^39,40^. Yan et al. showed that in MCF-7 cells overexpression of BRCA1 resulted in arrest at the G2/M stage of cell cycle^39^. Research by Razandi et al. revealed that, BRCA1 inhibited estrogen and growth factor receptor signaling to cell growth in breast cancer. They disclosed that proliferation of MCF-7 cells in response to estradiol (E2) was significantly inhibited by expression of wild type BRCA1 induced by lipofectamine transfection. Like p53, BRCA1 also upregulates ERK phosphatase function via the MKP-1 protein, which is vital in hindering E2-induced ERK and cellular proliferation. In addition, the intact tumor suppressor inhibited action of cyclin-dependent kinases CDK4 and CDK1, and paused cell cycle advancement at G1/S and G2/M phases^40^.

Our group previously demonstrated the molecular formula of carbonate apatite nanoparticles (CA NPs) is Ca_10_ (PO_4_)_6−x_ (CO_3_)_x_ (OH)_2_, containing PO_4_^3−^, CO_3_^2−^, OH^-^ groups on its surface which accounts for its negative zeta potential. Even though the particles have net negative charge, they are capable of binding anionic DNA apparently through the Ca^2+^ rich domains instead of the PO_4_^3−^ rich sites present on their surfaces ^21^. Hence, the positively charged Ca^2+^ in the CA NPs can electrostatically associate with the negatively charged plasmid DNA molecules and the resulting complex crosses the cell membrane via endocytosis^22^. Distinct from liposome mediated gene delivery, in our study we used inorganic CA nano vector for delivering the BRCA1 and BRCA2 genes into three different types of breast cancer cell lines. In MCF-7 cell line, reduced cell growth upon BRCA1 and BRCA2 gene delivery supports the finding of the previously reported results by Yan et al. and Razandi et al.^39,40^. In MCF-7 cells, we also found that successful expression of BRCA1 and BRCA2 lowered expression of P-MAPK in the MAPK signaling and controlled breast cancer cell proliferation. Similar to the results revealed by Razandi et al.; in our study, NP- facilitated delivery and successful expression of BRCA1 and BRCA2 might induce upregulation of MAPK phosphatase activity via the MKP-1 protein and lowered P-MAPK level detected in the lysates of MCF-7 cells treated with BRCA1 + NP and BRCA2 + NP.

In parallel to the cell viability results in murine 4T1 cell line, treatment with gene therapeutics (BRCA1 + NP and BRCA2 + NP) in syngeneic mouse model carrying 4T1-induced breast tumors, showed significant tumor regression pattern in comparison to the NP control group. The tumor volume, weight and tumor inhibition rate in BRCA1 + NP and BRCA2 + NP treatment groups were significantly lowered, indicating role of wild type BRCA1 and BRCA2 in the tumor microenvironment. Thus, considering our in vitro and in vivo findings, BRCA1- and BRCA2-based gene therapy using CA NPs as delivery vector reveals a highly promising approach to treating breast cancer. Thus, restoration of BRCA1/2 tumor suppressor activities through intracellular delivery of BRCA1/2 gene with the aid of CA NPs, suggestively repressed breast tumor growth both in vitro and in vivo, supporting the concept that BRCA1/2 might be the possible targets for gene-based therapy of breast cancer. Moreover, our research shed light on the possible applications of the pH-responsive NPs as an influential tool for DNA delivery in clinical settings in future.

## Materials and methods

### Materials

Dulbecco’s Modified Eagle Medium (DMEM) powder with high glucose (without sodium bicarbonate) and DMEM liquid were procured from Sigma (USA). Fetal bovine serum (FBS), trypsin-ethylene diamine tetra acetate (Trypsin–EDTA) and penicillin–streptomycin were purchased from Gibco BRL (California, USA); sodium bicarbonate, sodium sulphite, calcium chloride dihydrate (CaCl_2_.2H_2_O), dimethyl sulphoxide (DMSO) and thiazolyl blue tetrazolium bromide (MTT) reagent were bought from Sigma-Aldrich (St Louis, MO, USA). The plasmids encoding BRCA1 (catalog no. 61586) and BRCA2 (catalog no. 16245) tumor suppressor genes were ordered from ‘Addgene’ (USA), where _pc_BRCA1-385 was a gift from Lawrence Brody and _p_CIN BRCA2 WT was a gift from Mien-Chie Hung^41,42^. The antibiotic, ampicillin (Fisher Scientific), Luria Bertani (LB) agar from Merck (Germany) and LB broth were procured from Laboratorios CONDA (Spain). Two human breast cancer cell lines MCF-7, and MDA-MB-231, one mouse breast cancer cell line 4T1 were obtained from ATCC (American Type Culture Collection, Manassas, VA, USA). For Western Blotting, Tween-20 and Pierce ECL Western blot detection reagents were purchased from Bio-Rad Laboratories, Inc. (Hercules, CA, USA). Dithiothreitol (DTT) (Bio-Rad), bovine serum albumin (BSA) (Merck, USA), protease inhibitor and phosphatase inhibitor cocktail were obtained from Sigma. Monoclonal IgG primary antibodies raised in rabbit for phospho-MAPK, total MAPK, GAPDH, and the horseradish peroxidase (HRP)-conjugated secondary goat anti-rabbit IgG antibody were from Cell Signaling Technology, Inc. (Beverly, MA, USA).

### Methods

All methods were carried out in accordance with relevant guidelines and regulations.

### Formulation of CA NPs

DMEM powder (with high glucose and without sodium bicarbonate) was mixed with 44 mM of sodium bicarbonate and pH was adjusted to 7.4. Subsequently, 1 M exogenous CaCl_2_$\cdot$2H_2_O (4 μL for in vitro and 8 μL for in vivo usage) was added in the DMEM liquid, facilitating formation of CA nanoparticles as described elsewhere^20^. Next, BRCA1 and or BRCA2 plasmid was added in different concentrations to make 1 mL final volume of the fresh media, and incubated at 37 °C for 30 min, followed by addition of 10% FBS to the vial to prevent further generation and consequential aggregation of the particles. Addition of FBS was skipped in case of CA NPs for intravenous delivery of plasmids, and the formulation was kept on ice to prevent aggregation of particles^43^.

### Isolation of plasmid DNA for gene transfection

*Escherichia coli* DH5α bacteria harboring BRCA1 gene insert in pcDNA3-BRCA1 plasmid (5600 bp) or BRCA2 gene insert in pCIN-BRCA2 plasmid (10,000 bp) were grown in LB agar plates in presence of ampicillin (100 μg/mL) overnight at 37 °C. Distinct colonies were inoculated in 100 mL of LB broth in presence of ampicillin antibiotic and grown-up in an incubator at 220 rpm for 16–18 h at 37˚C. The plasmids were isolated and purified using plasmid Mega kit (Qiagen, Germany). The concentration of extracted plasmid DNA was measured in ‘Implen’ nanodrop machine. A ratio of absorbance at 260 and 280 with 1.8 reading or more was suggestive of highly pure extracted DNA. Furthermore, purified DNA was run on 0.8% agarose gel and the DNA bands were pictured under ultraviolet transilluminator using ‘Quantity One’ Bio-Rad software^43,44^.

### Size and zeta potential measurement

The size and zeta potential of BRCA1/BRCA2 plasmid complexed with CA NPs were measured using Zeta Sizer (Malvern, Nano, ZS, UK). The NPs, BRCA1 + NPs and BRCA2 + NP complexes were formed by adding 4 μl of 1 M CaCl_2_, and 1 μg of BRCA1 plasmid to make 1 mL of DMEM (pH 7.4), followed by incubation at 37 °C for 30 min. Next, 10% FBS was added to stop additional formation of NPs complexes which were maintained on ice till measurement was done with Zetasizer. The data was analyzed using Zetasizer software 6.20 and all samples were measured in duplicates, with the values presented as mean ± SD^44^.

### Cell culture and seeding

The three breast cancer cell lines—MCF-7, MDA-MB-231 and 4T1 were grown in culture flask in DMEM supplemented with 10% FBS and 1% penicillin and streptomycin antibiotic in a 37 °C incubator humidified with 5% CO_2_. One day prior to treatment, cells were trypsinised, centrifuged and resuspended using DMEM. Next, using a hemocytometer, cells were counted under an optical microscope and seeded in a 24-well plate with cell density of 50,000 cells per well. Before transfection, cells were allowed to attach overnight at 37 °C with 5% CO_2_^43^.

### Cell viability assessment with MTT assay

Following incubation of the breast cancer cells in presence of NPs, BRCA1 + NPs and BRCA2 + NPs for 48 h, 50 µL of MTT solution (5 mg/mL in PBS) was added to each well and incubated for 4 h at 37 °C. Next, the liquid medium was removed, soaked onto Kimwipe and 300 µL of DMSO was added to each well to dissolve the purple formazan crystals and absorbance was measured in a micro plate reader at 595 nm with a reference wavelength of 630 nm. Cell viabilities were normalized to the absorbance of untreated cells (control). Each treatment experiment was performed in triplicate and cell viability was expressed as mean ± SD. The cell viability in the treated wells was expressed as a percentage and calculated using the absorbance values attained from MTT assay^43^. The following formula was used to calculate the cell viability and cytotoxicity data:

1 $$\mathrm{Cell\, viability }\left(\mathrm{\%}\right)= \frac{\mathrm{Abs}\left(\mathrm{sample}\right)-\mathrm{Abs }(\mathrm{negative\, control})}{\mathrm{Abs }\left(\mathrm{positive\, control}\right)-\mathrm{Abs }(\mathrm{negative\, control})} \times 100$$

2 $$\% \;{\text{of}}\;{\text{cytotoxicity}} = {1}00 - \% \;{\text{of}}\;{\text{cell}}\;{\text{viability}}$$

3 $$\% \;{\text{of}}\;{\text{cytotoxicity}}\;\left( {{\text{for}}\;{\text{plasmid}}} \right) = \left( {{\text{Cytotoxicity}}\;{\text{for}}\;{\text{plasmid}} + {\text{NPs}}} \right) - \left( {{\text{Cytotoxicity}}\;{\text{for}}\;{\text{NPs}}} \right)$$

### Western blotting

MCF-7 cells were incubated with respective BRCA1/BRCA2 plasmid-loaded NPs for a consecutive period of 48 h and washed with a cool phosphate buffered saline prior to lysis. The cell lysates were centrifuged at 13,000 rpm for 10 min at 4 °C (Sartorius Stedim Biotech, Germany) and protein concentration of the cell lysate was estimated using the Bradford assay. The protein extracts were run with sodium dodecyl sulphate polyacrylamide gel electrophoresis (precast gel) at 60 V for 90 min and the determined proteins were electro-transferred for 7 min at 25 V to PVDF membranes (Thermo Scientific, USA) using Turbo transfer unit (Bio-Rad). The membranes were blocked for 1 h at room temperature with Tris buffered saline with Tween 20 (TBST) containing 5% skimmed milk (Merck, USA). The membranes were then incubated with 1:1000 primary antibody against Phospho-p44/42 MAPK or Total MAPK (Cell Signaling Technology, USA) in TBS-T with 5% bovine serum albumin for overnight, at 4 °C with slow rocking. The membranes were washed 5 times with TBST and incubated with 1:3000 secondary antibody (anti-rabbit IgG-HRP linked) for 60 min at room temperature with slow rocking. Next, Clarity Western ECL substrate (Bio-Rad) was added following incubation for 5 min. Bands were visualized using BioRad Gel Doc system with Quantity One chemiluminescent software. Image J software was used to quantify the bands on the membranes^43^. The original Western blot images are provided in the supplementary information below.

### Study of tumor regression pattern in a breast tumor-induced mouse model (in vivo)

The 4T1 is a transplantable tumor cell line, highly tumorigenic and can easily metastasize^45^. The tumor development and metastatic spread of 4T1 cells in Balb/c mice very likely mimic human breast cancer^46^. In mammary tumor model, 4T1 cells-induced breast cancer mouse models are the most common type for investigating antitumor effect of various therapeutic formulations^47^. In our study, female Balb/c mice (6–8 weeks old) of 15–20 gm of body weights were purchased from School of Medicine and Health Science animal facility, Monash University and maintained in 12:12 light: dark condition by providing them ad libitum chow and water. All experiments were done in accordance with the regulations imposed by Monash University Animal Ethics Committee, with animal ethics approval no. MARP/2016/126.

Approximately 1 × 10^5^ 4T1 cells (in 100 µL PBS) were injected subcutaneously using 27 gauge needle on the mammary pad of mice unilaterally (considered as day 1) and the mice were checked between 3 and 5 days for the development of tumor by touching the area of injection with index finger. As the tumor volume reached around 25–30 mm^3^ , the mice were randomly distributed into three different groups (5 mice per group) for treatments^43^. Mice were treated intravenously through tail-vein injection with 100 μL of NP (8 M Ca^2+^), BRCA1 + NP (40 µg of plasmid) and BRCA2 + NP (40 µg of plasmid). The second dose was administered at day 10, i.e., after 2 days from the 1st dose. During the treatment period, the size of the tumor was measured at regular intervals (4 times/week). The gross body weights of mice were monitored and the lengths and widths of the tumors were measured using the vernier caliper in mm scale for 22 days. Also the gross body weights of mice and their activities were monitored for study purpose. The volume of the tumor was calculated using the following formula:

$$\mathrm{Tumour \; volume }\left({\mathrm{mm}}^{3}\right)=\frac{1}{2}(\mathrm{Length }\times {\mathrm{Width}}^{2})$$

The data are presented here as the mean ± SD of tumor volumes from each group. At the end of the study, at day 22, mice were humanly sacrificed by cervical dislocation following 100% CO_2_ exposure for few seconds and tumors were excised for further study^43^.

### Statistical analysis

For cell viability study, data were expressed as means ± standard deviation (SD) for (n = 3) biological replicates. Student's t-test was used all through. For in vivo tumor regression study, LSD post-hoc test for one-way ANOVA was used to analyze and compare the significant difference between treatment groups^43^. The differences among groups were considered significant at *p* < 0.05.

### Institutional review board statement

The animal study was approved by the Monash University Animal Ethics Committee, with animal ethics approval no. MARP/2016/126.

### ARRIVE guidelines statement

The authors have read the ARRIVE guidelines, and the study was reported according to the ARRIVE guidelines (Supplementary Information).

## Supplementary Information

Supplementary Information.

## Data Availability

The datasets regarding the plasmids used in the current study are available in the following links: https://www.addgene.org/61586/ and https://www.addgene.org/16245/.
