# Supplementary material for: Retarding breast tumor growth with nanoparticle-facilitated intravenous delivery of BRCA1 and BRCA2 tumor suppressor genes
Source: Sci Rep. 2023 Jan 11;13:536. doi: 10.1038/s41598-022-25511-9 (PMC9834397; doi:10.1038/s41598-022-25511-9)
Supplement: Supplementary file 1 — Supplementary Information. [file 41598_2022_25511_MOESM1_ESM.pdf]

Title: Retarding breast tumor growth with nanoparticle-facilitated intravenous delivery of BRCA1 and BRCA2 tumor suppressor genes

Nabilah Ibnat[1,2], Ezharul Hoque Chowdhury[1]*

[1] Jeffrey Cheah School of Medicine and Health Sciences, Monash University Malaysia, Selangor, Malaysia.

[2] Department of Bioengineering, George Mason University, Fairfax, Virginia, 20110.

* Correspondence: md.ezharul.hoque@monash.edu; Tel: +603 5514 5628.

Original Western Blot Images for BRCA1+NP, BRCA2+NP treatments

1	2	3	4	5	6	7	8	9	10	11	12	13	14	15

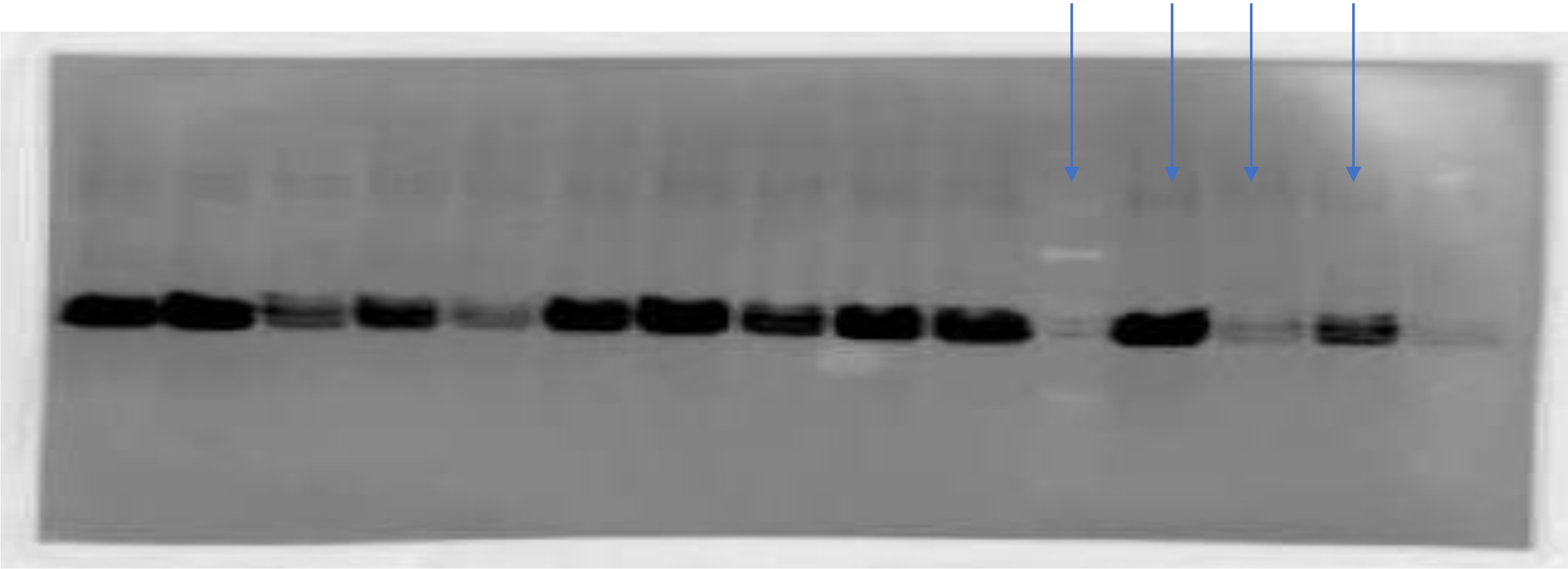

Lane 1-10: Other samples

Lane 11: Ladder
Lane 12: NP
Lane 13: BRCA1+NP
Lane 14: BRCA2+NP

Fig. Original Western Blot Image for Phospho-MAPK (P-MAPK)

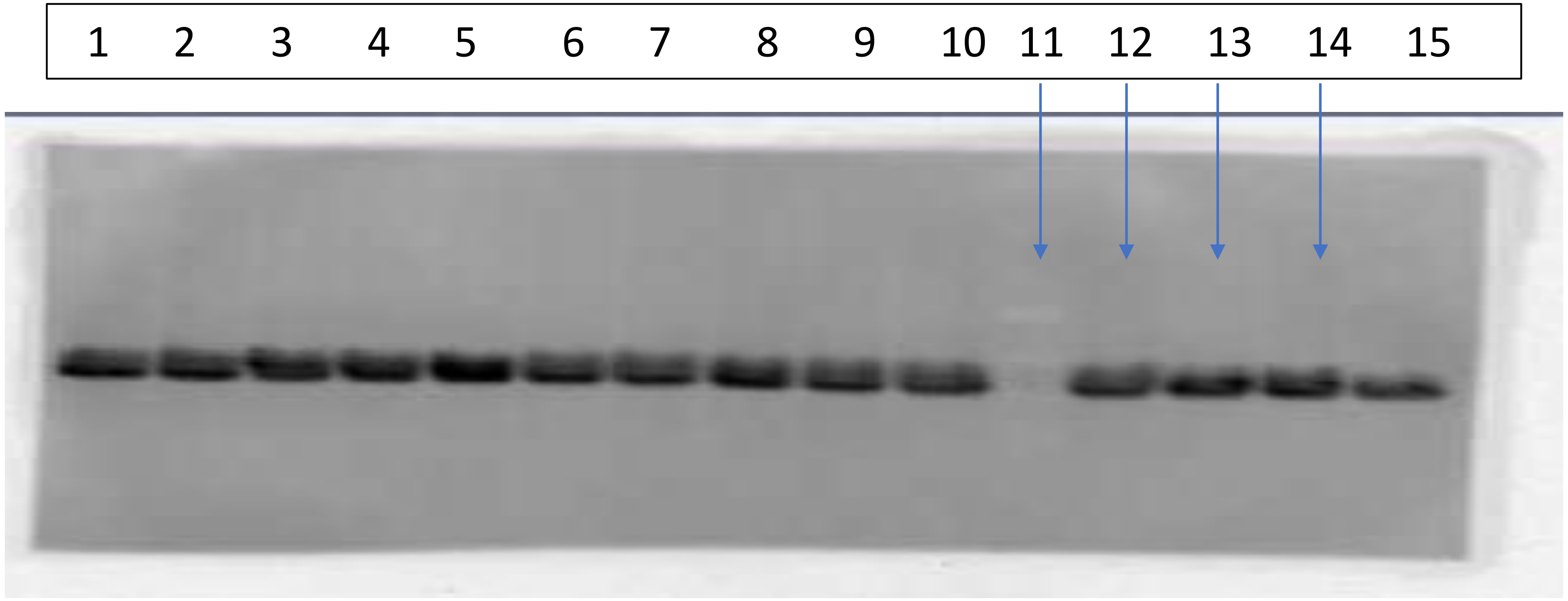

Lane 1-10: Other samples

Lane 11: Ladder
Lane 12: NP
Lane 13: BRCA1+NP
Lane 14: BRCA2+NP

Fig. Original Western Blot Image for Total-MAPK (T-MAPK)

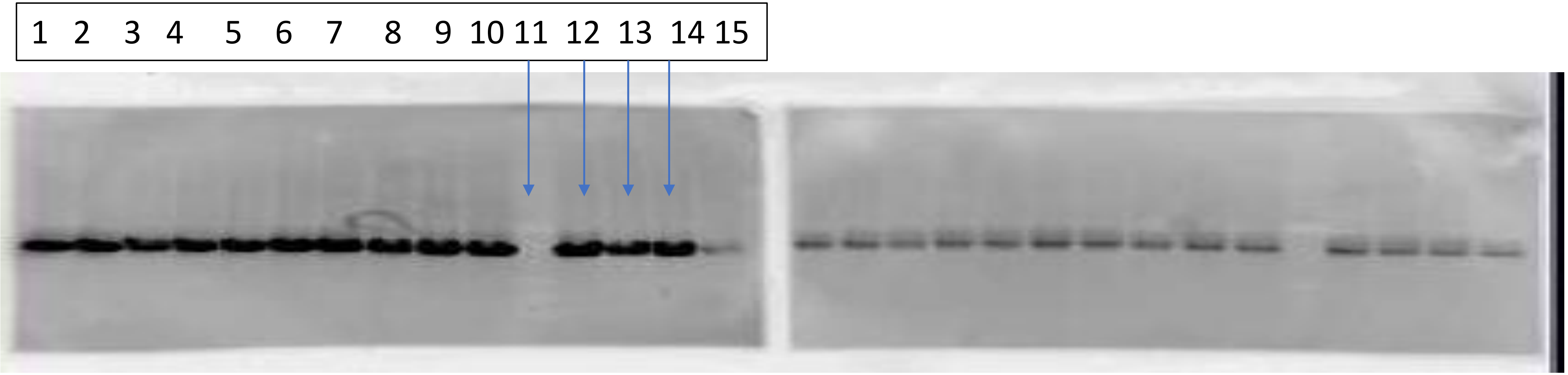

Fig. Original Western Blot Image for GAPDH

Lane 1-10: Other samples

Lane 11: Ladder
Lane 12: NP
Lane 13: BRCA1+NP
Lane 14: BRCA2+NP
